# MASTL induces Colon Cancer progression and Chemoresistance by promoting Wnt/β-catenin signaling

**DOI:** 10.1186/s12943-018-0848-3

**Published:** 2018-08-01

**Authors:** Srijayaprakash Babu Uppada, Saiprasad Gowrikumar, Rizwan Ahmad, Balawant Kumar, Bryan Szeglin, Xi Chen, J. Joshua Smith, Surinder K. Batra, Amar B. Singh, Punita Dhawan

**Affiliations:** 1 Department of Biochemistry and Molecular Biology, University of Nebraska Medical Center, Omaha, NE-68022 USA; 2 Buffet Cancer Center, University of Nebraska Medical Center, Omaha, NE USA; 3 Department of Surgery, Colorectal Service, Memorial Sloan Kettering Cancer Center, New York, NY USA; 4 Human Oncology and Pathogenesis Program at MSKCC, New York, NY USA; 5 Division of Biostatistics, University of Miami Miller School of Medicine, Miami, FL USA; 6 VA Nebraska-Western Iowa Health Care System, Omaha, NE USA

**Keywords:** Colon cancer, Wnt signaling and MASTL

## Abstract

**Background:**

Chemotherapeutic agents that modulate cell cycle checkpoints and/or tumor-specific pathways have shown immense promise in preclinical and clinical studies aimed at anti-cancer therapy. MASTL (Greatwall in Xenopus and Drosophila), a serine/threonine kinase controls the final G2/M checkpoint and prevents premature entry of cells into mitosis. Recent studies suggest that MASTL expression is highly upregulated in cancer and confers resistance against chemotherapy. However, the role and mechanism/s of MASTL mediated regulation of tumorigenesis remains poorly understood.

**Methods:**

We utilized a large patient cohort and mouse models of colon cancer as well as colon cancer cells to determine the role of Mastl and associated mechanism in colon cancer.

**Results:**

Here**,** we show that MASTL expression increases in colon cancer across all cancer stages compared with normal colon tissue (*P* < 0.001). Also, increased levels of MASTL associated with high-risk of the disease and poor prognosis. Further, the shRNA silencing of MASTL expression in colon cancer cells induced cell cycle arrest and apoptosis in vitro and inhibited xenograft-tumor growth in vivo. Mechanistic analysis revealed that MASTL expression facilitates colon cancer progression by promoting the β-catenin/Wnt signaling, the key signaling pathway implicated in colon carcinogenesis, and up-regulating anti-apoptotic proteins, Bcl-xL and Survivin. Further studies where colorectal cancer (CRC) cells were subjected to 5-fluorouracil (5FU) treatment revealed a sharp increase in MASTL expression upon chemotherapy, along with increases in Bcl-xL and Survivin expression. Most notably, inhibition of MASTL in these cells induced chemosensitivity to 5FU with downregulation of Survivin and Bcl-xL expression.

**Conclusion:**

Overall, our data shed light on the heretofore-undescribed mechanistic role of MASTL in key oncogenic signaling pathway/s to regulate colon cancer progression and chemo-resistance that would tremendously help to overcome drug resistance in colon cancer treatment.

**Electronic supplementary material:**

The online version of this article (10.1186/s12943-018-0848-3) contains supplementary material, which is available to authorized users.

## Background

Loss of cell-cycle control, a key regulatory aspect of normal growth, is a hallmark of neoplastic growth and malignancy, including in CRC. It is remarkable that due to the deregulation of cell cycle control, cancer cells evade programmed cell death despite accumulation of the genomic instabilities that would normally make them prime targets for apoptosis and cause them to divide rapidly. Unfortunately, currently available therapeutic drugs aimed at controlling the cell cycle in cancer cells have lacked the therapeutic index required to achieve a robust response against cancer cells while having little or no cytotoxic effect on normal cells. Thus, one of the strategy might be to target cell-cycle regulatory features distinctive to tumor cells.

In this regard, cell cycle kinases play a key role in promoting cell cycle progression through its different phases. Among these kinases, MASTL (named Greatwall in Xenopus and Drosophila) was identified recently and is now demonstrated to be important for mitosis, especially the G2/M checkpoint. More specifically, MASTL kinase activity prevents cells from premature entry into mitosis, and therefore minimizes chromosomal mis-segregation. To promote the G2/M transition, MASTL inhibits PP2A activity by phosphorylating ARPP19 and a-endosulfine (ENSA). As would be expected, genetic depletion of MASTL in young mice compromised survival, and this was due to severe proliferation defects [1]. MASTL expression, however, also helps to regulate recovery following DNA damage and inhibiting MASTL has been demonstrated to be beneficial for DNA damage-based therapies [2]. In line with known significance of these traits in malignant growth, upregulated expression of MASTL has been reported in breast, head, and neck cancers and is correlated with aggressive clinico-pathological features [2]. Moreover, a causal role for MASTL in resistance against anti-cancer therapies has been demonstrated using cell lines derived from initial and recurrent tumors of head and neck squamous cell carcinoma [3]. These studies suggest a critical role for MASTL in oncogenic growth and tumorigenesis. However, a causal association of MASTL in regulating colon cancer growth and progression and its potential role in resistance to conventional therapy, a critical factor in unrelenting patient death, remains an area of active investigation.

In this study, we demonstrate, using a comprehensive investigative scheme, a significant upregulation of MASTL expression in stage-specific manner in CRC progression and an inverse correlation with patient survival. We further show its causal significance in cancer progression and resistance to anti-CRC therapy. Mechanistically, we provide strong evidence for a novel role for MASTL in regulating Wnt/β-catenin signaling to modulate c-Myc and Survivin expression in promoting colon cancer. Overall these data identify MASTL as a novel therapeutic target in limiting colon cancer malignancy and reducing death from the disease.

## Methods

### Cell culture, plasmids and transfection

The human colon cancer cell lines HCT116, SW620, SW480, HT29, DLD-1, CaCo2, Ls174T, and IEC-6 cells were obtained from ATCC (Manassas, VA, USA) and cultured in RPMI-1640 containing 10% fetal bovine serum and 1% antibiotic and antimycotic (thermoFisher). Cells were transfected as described previously using effectene reagent [4]. Mastl-knockdown cell population was selected using puromycin (1 mg/ml). The activated β-catenin (S33Y) mutant was described previously [5].

### Human tissue, microarray platforms and statistical analysis

RNA from human samples was hybridized to Affymetrix Human Genome U133 Plus 2.0 GeneChip Expression Array.The protocols and procedures for the procurement of human tissue samples and details of the microarray platforms and statistical analysis have been described previously [6, 7].

### Immunoblot, immunohistochemistry and immunofluorescence analysis

These analyses were performed using the standard protocols as described before [4]. Anti-MASTL Antibody (clone 4F9, MABT372, EMD Millipore), anti-E-cadherin antibody (BD transduction laboratories, USA), β-catenin (BD transduction laboratories, USA), GSK3beta (Cell Signaling Technology, Danvers, MA,USA), p-GSK3beta (Cell Signaling Technology, Danvers, MA,USA) Bcl-xL (Cell Signaling Technology), Survivin (Cell Signaling Technology) and anti-b- actin (Sigma, St. Louis, MO), were used for immunoblotting.

### Cell proliferation MTT assay and soft agar assay

To assess cell proliferation, MTT assay was performed as described previously [7]. Anchorage-independence growth assay were used to determine the growth potential of MASTL knockdown cells as described previously [7].

### Oncogenic Array

Oncogenic array analysis was performed using proteome profiler human xl oncology array kit (R&D Systems, Minneapolis, MN)) as per manufacturer’s instructions.

### Invasion assay

Invasive potential of cells was measured in transwell filter insert with 8.0 μm pore polycarbonate membrane (Corning) coated with Matrigel (BD, Franklin Lakes, NJ, USA) as described previously [7].

### Edu proliferation

The 5-ethynyl-2′-deoxyuridine (EdU), a thymidine analogue, is incorporated into cellular DNA during DNA replication [8]. The incorporated EdU can be detected through a reaction between ethynyl group of EdU and a fluorescent azide in a copper-catalyzed [3 + 2] cycloaddition (“Click” reaction) using Click-iT™ EdU imaging kit (Invitrogen, Carlsbad, CA) as per manufacturer protocol.

### Caspase-3 activity assay

CaspACE™ Assay System (Promega Corp., Madison, WI) was used to detect caspase-3 activity as per manufacturer protocol.

### Annexin V-fluorescein isothiocyanate/ propidium iodide staining

We used the Hoechst/annexin V-fluorescein isothiocyanate (FITC)/ propidium iodide (PI) triple staining detection system to assess cell apoptosis. FITC Annexin V Apoptosis Detection Kit II (BD Biosciences, San Jose, CA) was used as per the manufacturer’s instructions.

### RNA extraction and real-time RT-PCR

Total RNA was extracted using RNeasy Plus Mini Kit (QIAGEN) according to manufacturer instructions as described [7].

### Cell cycle analysis

Transfected cells were harvested and plated in six-well plates and cultured for 72 h in serum-free medium after which cells were treated with RO3306 (Sigma, St. Louis, MO), a CDK1 inhibitor for 16 h. After 16 h, media was replaced with fresh media and cells were grown for 1 h, and then fixed and cell cycle analysis was carried out. The percentage of cells in G0/G1, S, and G2/M phases of the cell cycle was determined using flow cytometer (FACS Calibur, BD Biosciences, San Jose, CA) after PI staining.

### Xenograft-tumor studies

All animal experiments were conducted with the approval of the Institutional Animal Care and Use Committee (IACUC) of UNMC. The tumorigenicity of cells under study was assessed using subcutaneous flank inoculation of 1 × 10^6^ cells in 6-week-old athymic nude mice. Animals were assessed for 5 weeks after the inoculation for tumor incidence and growth and then were sacrificed Tumor volume was measured using the formula Tumor volume = 1/2(length × width^2^)/2 as previously described [2, 7].

### Statistical analysis

Statistical analyses were performed using Graphpad Prism software (San Diego, CA) for t-test analysis, where comparisons between two groups were involved, and analysis of variance were, more groups are present to determine statistical significance, and differences were considered statistically significant at *P* < 0.05.

## Results

### MASTL is markedly upregulated in colorectal cancer

**Fig. 1:**
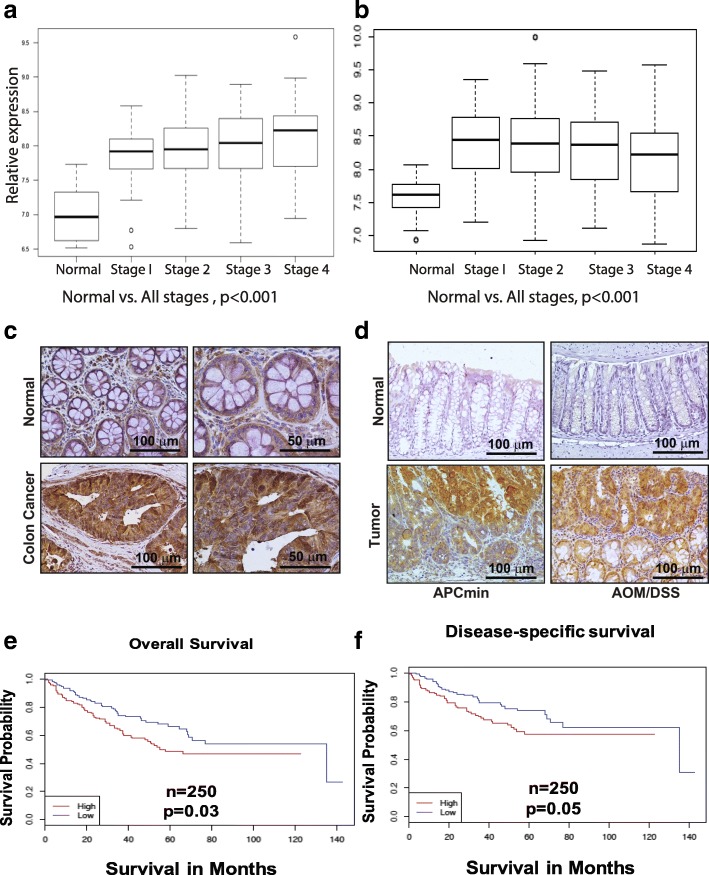
MASTL expression is upregulated in colon cancer. Patients were evaluated by tumor stage and expression levels were compared to expression levels of normal adjacent samples. **a** 250 patients were analyzed from the VMC/MCC data set. Wilcoxon rank sum test was used to test for significance between each stage and normal (*P* < 0.001). **b** 315 patients were analyzed from the TCGA database. Kruskal- Wallis rank sum test was used to test for significance between each stage and normal (*P* < 0.001). Immunohistochemical analysis of (**c**) normal and colon cancer patients & (**d**) AOM/DSS and APC^min^ mice tumors for MASTL expression. **e** & **f** Comparison of overall survival and disease-specific survival in correlation with MASTL expression. Patients with high expression were defined as having greater than median expression of MASTL and were compared to the low expression group (less than the median expression value). Kaplan-Meier analysis was performed, comparing patients with high MASTL expression (red line) to low MASTL expression (blue line). Higher expression of MASTL correlated with significantly worse overall survival (*P* = 0.03, *n* = 250) (A) and a trend toward worse disease-specific survival (*P* = 0.05, *n* = 250)

To characterize the potential role of MASTL in colon carcinogenesis, we first assessed its expression in a high through-put transcriptome analysis of a large patient cohort (combined Moffitt Cancer Center/Vanderbilt Medical Center expression array data set using 250 CRC patient tumors and 10 normal adjacent tissue samples as described previously [demographics; [7]]). We found robust stage-specific up-regulation of MASTL transcript levels compared to normal adjacent mucosal specimens (Fig. 1a; *P* < 0.001). We found a similar significant increase in MASTL expression in all stages of colon cancer compared to normal samples, analyzing the TCGA database (Fig. 1b).

To examine if the observed increase in MASTL mRNA expression was translationally relevant, we determined MASTL expression in a panel of colorectal cancer cell lines, animal models of colon cancer as well as a commercial colon cancer tissue array [immunohistochemical (IHC) analysis; 50 samples]. We found that immunoblotting using lysates from confluence matched cell lines indeed demonstrated a similar increase in MASTL expression in colon cancer cells versus non- transformed intestinal epithelial cells (IEC-6) (Additional file 1: Figure S1A). IHC analysis also revealed marked increase in MASTL expression in CRC tissue samples (compared to normal colon; Fig. 1c). To corroborate these findings, we further determined MASTL expression in colon tumor samples from a murine model of sporadic colon cancer (CRC; the APCmin mice) and colitis- associated cancer (CAC; AOM/DSS induced mouse model). A significant increase in MASTL expression was observed in tumors from both colon cancer models (Fig. 1d). These findings validated a positive association between MASTL expression and colon cancer progression.

We further determined whether high MASTL expression could also identify high-risk colon cancer patients. Overall survival estimates based on MASTL expression were determined in the CRC patient database as described previously [7]. We used a median cut-off for MASTL expression (higher-than-median = high MASTL expression; lower-than-median = low MASTL expression). We noted a significant association of better overall survival for patients with lower-than-median MASTL expression (Fig. 1e, *p = 0.03*) while patients exhibiting high MASTL expression had worse overall survival. We found a similar trend using disease-specific survival as an outcome measure (Fig. 1f, *p* = 0.05). Our additional analysis, wherein we divided patients into four quartiles based on MASTL expression values and performed Kaplan-Meier analysis, revealed a similar trend (Additional file 1: Figure S1B). These data rendered strong evidence that MASTL can serve as a prognostic biomarker for latent disease aggressiveness among colorectal cancer patients.

### Inhibiting MASTL expression in colon cancer cells inhibits neoplastic growth and invasive mobility

**Fig. 2:**
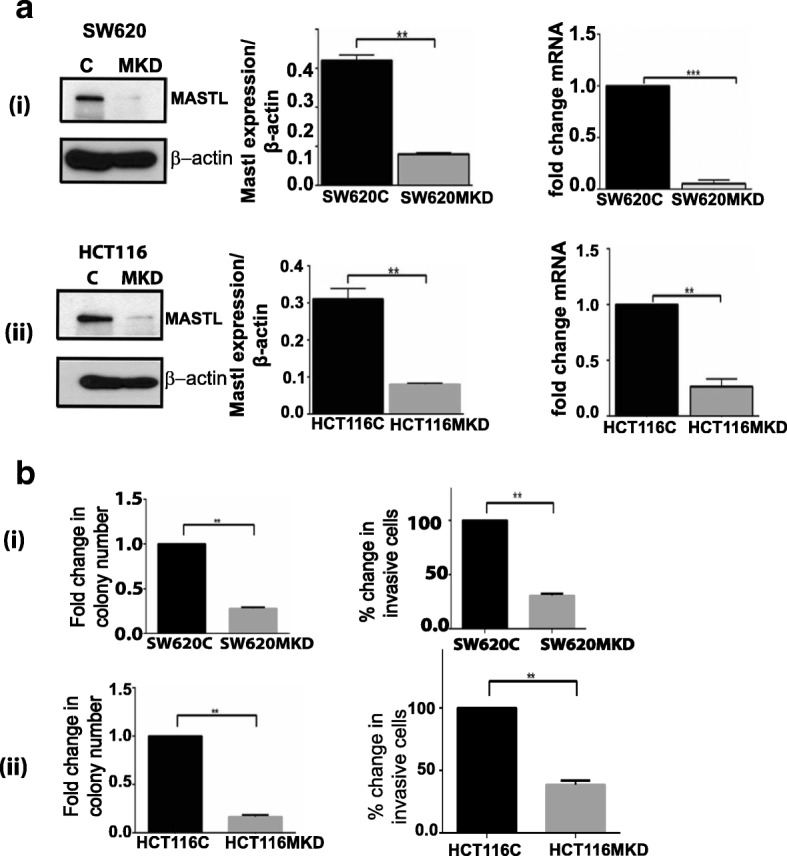
MASTL knockdown results in altered functional characteristics in colon cancer cell lines in vitro. **a** (i) MASTL knockdown in SW620 cells was confirmed by Western blot and qRT-PCR analysis. (ii) MASTL knockdown in HCT116 cells was confirmed by Western blot and qRT-PCR analysis. **b** Tumorigenic and invasive potential was determined by ability to form colonies in soft agar assay and invasion in (i) SW620C and SW620^MKD^ cells (ii) HCT116C and HCT116^MKD^ cells. For graphs, data represent mean ± SD; **, *P* < 0.001

In further studies, to identify the causal significance of MASTL expression in CRC progression and the specific tumorigenic trait that is affected by MASTL expression, we evaluated tumorigenic and invasive properties of HCT116 and SW620 cells in response to inhibition of MASTL expression. Selection of cell lines for these studies was based on known tumorigenic/metastatic potential and high MASTL expression. Anti-human MASTL shRNA was expressed in these cells and silencing efficiency was confirmed by qRT-PCR, immunoblotting and immunofluorescence analyses (Fig. 2a (i&ii), Additional file 1: Figure S2). HCT116MKD and SW620MKD cells (with inhibition of MASTL expression) were analyzed using the anchorage-independent growth and matrigel-coated transwell-based invasion assays. Inhibition of MASTL expression significantly inhibited cell invasion (*P* < 0.05) and the ability of these cells to form colonies in soft agar by 60–80% (*P* < 0.05) {Fig. 2b (i&ii) and Additional file 1: Figure S3}. These data supported a necessary role of MASTL in promoting the oncogenic and metastatic properties of colon cancer cells.

### MASTL knockdown arrests cell cycle at G2/M and induces apoptosis in colon cancer cells

To ascertain specific cellular function affected by MASTL expression for these observed changes, we performed cell cycle analysis using HCT116MKD and SW620MKD and respective control cells.

Confluent cell monolayer was serum-starved for 72 h to achieve cell synchronization.

**Fig. 3:**
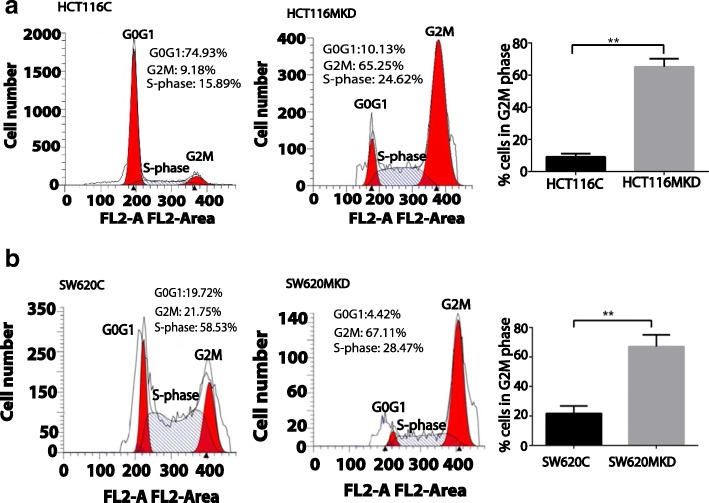
Cell are arrested in G2M phase as a result of MASTL knockdown in colon cancer. Control and MASTL knockdown cells were synchronized in serum free conditions for 72 h after which are treated with RO3306 for 16 h and then grown for one hour in fresh media. Cells were fixed immediately and cell cycle analysis was carried out via FACS. RO3306 is a selective ATP-competitive inhibitor of CDK1 that reversibly arrests proliferating human cells at the G2/M phase border, and the arrested cells enter mitosis rapidly after release from the G2 block [8] (**a**) HCT116 MASTL knockdown cells were unable to overcome the G2M block as compared to control cells (**b**) Similarly, the SW620 MASTL knockdown cells were also significantly arrested in G2M phase. For graphs, data represent mean ± SD; *, *P* < 0.01

Thereafter, cells were treated with RO3306 (10 μM), widely used to arrest cell cycle at G2/M interphase [8] for 16 h. Cells were released into the cell cycle by exposing them to fresh medium for 1 h. At this time, cells were collected for cell cycle analysis. As shown in Fig. 3, control cells progressed in the cell cycle to the G0/G1 phase. MASTL knockdown cells, however were unable to overcome the G2/M block by RO3306 so as to enter mitosis. As expected, beyond this, the percentage of cells in G2/M was 3–5 fold higher in HCT116MKD cells (Fig. 3a, *p* < 0.05) and SW620MKD cells (Fig. 3b, *p* < 0.05) compared to respective control cells.

**Fig. 4:**
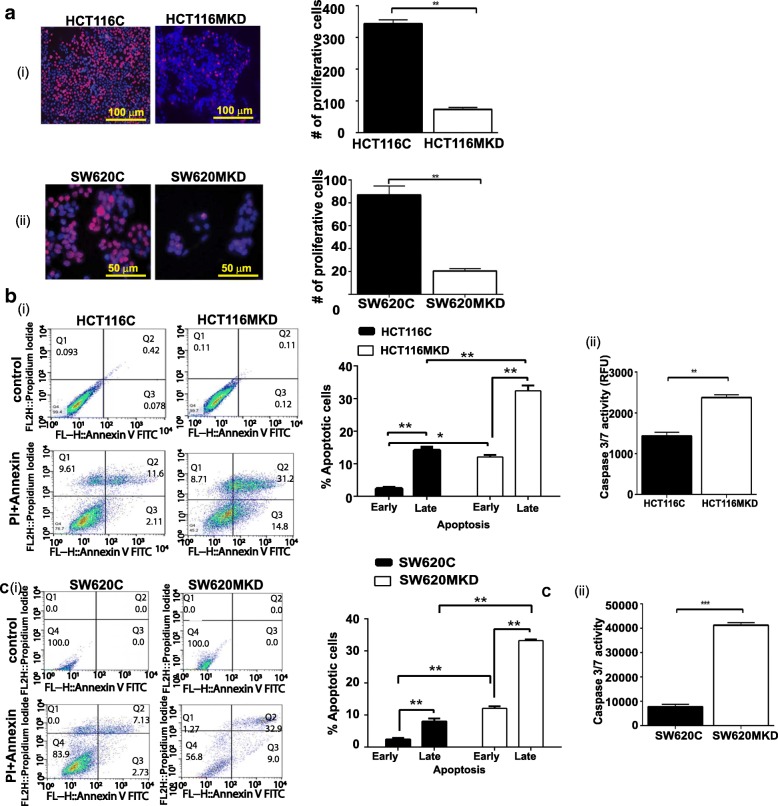
Cell cycle arrest induces apoptosis in MASTL inhibited cells. Control and MASTL knockdown cells were synchronized in serum-free conditions for 72 h, after which they were treated with RO3306 for 16 h. Media was replaced with fresh media for one hour after which cells were collected for (**a**) Edu cell proliferation assay and quantitation of number of proliferative cells; (**b** & **c**) (i) FITC Annexin V staining analysis via FACS. HCT116 and SW620 MASTL knockdown cells showed a significant induction of apoptosis at both early and late phases compared to control cells. (ii) Caspase activity assay further confirmed increased apoptosis in MASTL knockdown cells compared to control cells. For graphs, data represent mean ± SD; * *P* < 0.01

G2/M arrest can lead to apoptosis in various cancer cell lines, including colon cancer cells [9–11]. We thus next determined whether MASTL knockdown modulated proliferation or apoptosis by inhibiting the cell cycle at the G2/M interphase. To determine potential changes in cell- proliferation, MASTL knockdown and control cells were subjected to Edu-incorporation assay where it was observed that inhibiting MASTL expression significantly inhibited proliferative capacity of HCT116MKD and SW620MKD cells (Fig. 4a (i&ii)). To determine potential changes in apoptosis, cells were subjected to annexin-V and caspase activity assays. The annexin-V analysis revealed significant increases in both early and late apoptosis in HCT116MKD (Fig. 4b(i)) and SW620MKD cells (Fig. 4c(i)) compared to control cells (*p* < 0.05). Caspase 3/7 activity was also increased − 3-4 fold in these cells, suggesting a necessary role for MASTL in the regulation of processes determining cell multiplication and death (Fig. 4b (ii), C(ii)).

### MASTL regulates the anti-apoptotic proteins, Survivin and Bcl-xL, by modulating Wnt/β- catenin signaling to promote colon tumorigenesis

**Fig. 5:**
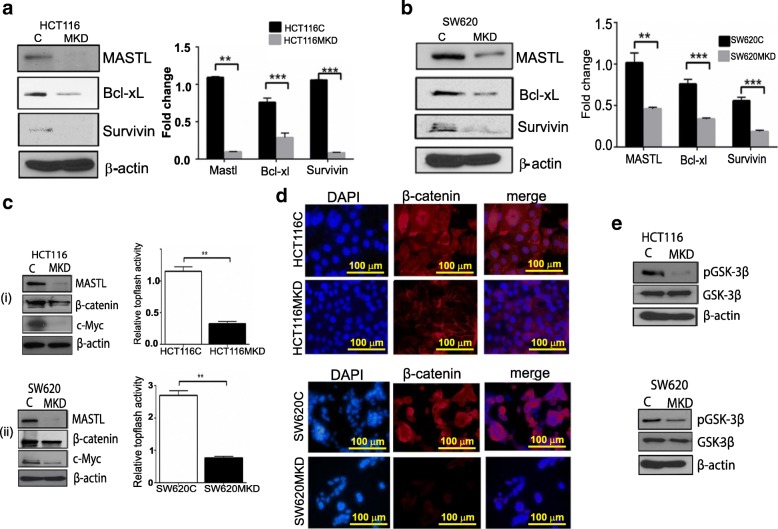
Anti-apoptotic proteins Bcl-xL and/or Survivin are downregulated in MASTL knockdown cells. (**a** & **b**) Bcl-xL and/or Survivin were downregulated with MASTL knockdown in both HCT116 and SW620 cells compared to control cells. For graphs, data represent mean ± SD;**, *P* < 0.001; ***, *P* < 0.0001 versus control. (**c** & **d**) β-catenin expression, activity and localization and c-Myc expression were altered in MASTL inhibited cells. (**e**) *p*-GSK-3β expression is altered with modulation of MASTL expression

To further examine the precise molecular mechanisms modified by genetic silencing of MASTL expression to induce aggressive phenotype, we performed an analysis of the global changes in protein expression, especially for proteins implicated in promoting oncogenesis, using a well-controlled and commercially available oncogenic array (Additional file 1: Figure S3). We focused on proteins which were reproducibly altered in both colon cancer cells (HCT116^MKD^ and SW620^MKD^). As shown in Additional file 1: Figure S4, we found consistent and significant alterations in the expression of Survivin and Bcl-xL, key proteins of anti-apoptotic pathways and upregulated in cancer cells [12–15], in MASTL-silenced cells versus control cells. We further confirmed significant downregulation of Bcl-xL and Survivin expressions using immunoblotting in MASTL-inhibited colon cancer cells compared to respective controls (Fig. 5a, b). Of note, Survivin is one of the downstream target genes of the Wnt/β-catenin signaling pathway [15, 16]. A critical significance of hyper-activated Wnt/β-catenin signaling in colon tumorigenesis is well established [16, 17]. Moreover, β-catenin expression is elevated during the G2/M interphase of the cell-cycle progression [18, 19]. We therefore reasoned that there might be a causal correlation between MASTL and Wnt/β-catenin signaling in promoting colon carcinogenesis through modulating Survivin and/or Bcl-xL expressions. Therefore, we further determined the effects of MASTL-knockdown upon β-catenin expression, cellular localization, and transcriptional activity (Fig. 5c (i&ii)). We found that knockdown of MASTL also resulted in sharp decreases in β-catenin expression, as well as expression of c-Myc, a Wnt/β-catenin signaling target gene. Further determinations demonstrated marked decreases in the nuclear accumulation of β- catenin and transcriptional activity, as measured by the TOP-Flash reporter activity, in MASTL inhibited colon cancer cells. In contrast, forced overexpression of full-length MASTL cDNA in colon cancer cells induced sharp increases in the expression of these proteins (Additional file 1: Figure S5). To see if similar correlation between these proteins existed in CRC patient samples, we interrogated expression levels of the c-Myc, Bcl-xL and MASTL in the 260-patient CRC database used to determine MASTL expression in CRC. It was quite encouraging that levels of c-Myc and Bcl-xL (BCL2L1) expression associated with MASTL in a similar fashion in patient samples as noted in vitro (Additional file 1: Figure S6).

Of importance, glycogen synthase kinase 3 (GSK3), in complex with Axin and *adenomatous polyposis coli* (APC), phosphorylates β-catenin at Thr41, Ser37, and Ser33. Phosphorylated β-catenin is specifically recognized by β-TrCP, a subunit of the SCF^β-TrCP^ E3 ubiquitin ligase complex. The SCF^β-TrCP^ ubiquitin ligase poly-ubiquitinates β-catenin, leading to β-catenin degradation via the proteosome pathway [20].

In contrast, phosphorylated and/or inactive GSK-3β promotes cellular accumulation and nuclear translocation of β-catenin, and initiation of the T-cell factor (Tcf)-dependent transcription. In agreement with this, GSK-3β phosphorylation (S9, inactive form) was seen to be markedly reduced in MASTL knockdown cells, resulting in active GSK-3β to induce β-catenin degradation (Fig. 5d). Further analysis showed no significant change in β-catenin mRNA expression, well in accordance with potential post-transcriptional regulation (data not shown).

### Inhibiting MASTL expression inhibits xenograft tumor formation by colon cancer cells in vivo

**Fig. 6:**
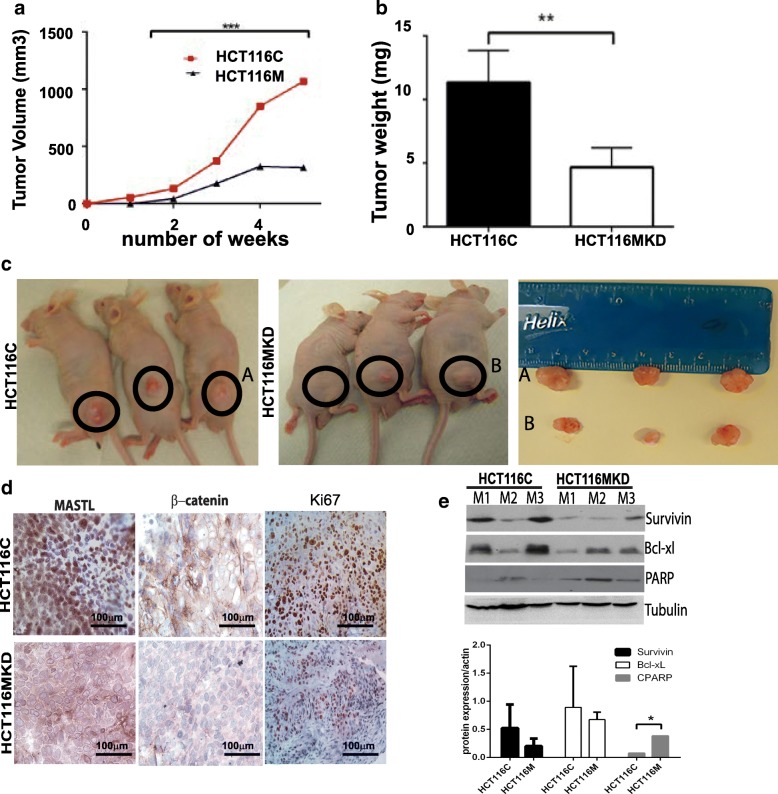
Effect of modulation of MASTL expression on tumor xenograft in vivo. (**a**-**c**) Flank tumor xenograft tumor development, after subcutaneous injection (*n* = 6 mice per group), was monitored for HCT116C, or HCT116MKD cells. Tumor volume and tumor weight of HCT116C and HCT116MKD cells after 4 weeks of inoculation in nude mice. (**d**) Tumors were evaluated for MASTL and β-catenin expression as well as markers of proliferation (Ki67) by immunohistochemistry; (**e**) Immunoblotting on tumors from 3 mice from each group (M1, M2 and M3) for Survivin, Bcl-xL and Cleaved PARP and normalized with tubulin as control. For graphs, data represent mean ± SD; **, *P* < 0.001; ***, *P* < 0.0001 versus control

To determine if inhibiting MASTL expression can similarly modulate colon tumorigenesis in vivo, we performed a subcutaneous xenograft tumor assay using HCT116MKD and respective control cells in athymic nude mice (*n* = 6/group). The same mice received control and MASTL knockdown cells on opposite flanks. In line with previous reports [7], mice receiving HCT116C cells demonstrated tumor development as early as two weeks post-injection of cancer cells, and average tumor volume was 1068 ± 161.2 mm3 at 4-weeks post-injection. By contrast, tumors resulting from injection of HCT116MKD cells were significantly smaller, with average volumes of 309 ± 50.6 mm3 after the same period of growth (Fig. 6a, c). Tumor weight followed a similar pattern and was lower (*P* < 0.05) in mice injected with MASTL-inhibited cells compared to those injected with control cells (Fig. 6b). Resulting tumors were then evaluated for expression of MASTL, β-catenin, Survivin, and Bcl-xL expression. Also effects of MASTL inhibition on cell proliferation, and apoptosis in tumors were determined (Fig. 6d, e). Similar to in vitro findings, MASTL inhibition reduced expression of β-catenin, Survivin and Bcl-xL expression in tumors resulting from HCT116MKD cells. Further, an increased rate of apoptosis, as determined by cleaved PARP expression, while decreased proliferation as determined by Ki67 immunoreactivity in tumors resulting from HCT116MKD was observed. This suggests that inhibiting MASTL expression restores a cell death program and inhibits proliferation. These data from xenograft tumor assays provide further support for the role of MASTL in tumorigenesis in CRC.

### Forced expression of genetically stabilized β-catenin rescues cellular apoptosis induced by inhibiting MASTL expression

**Fig. 7:**
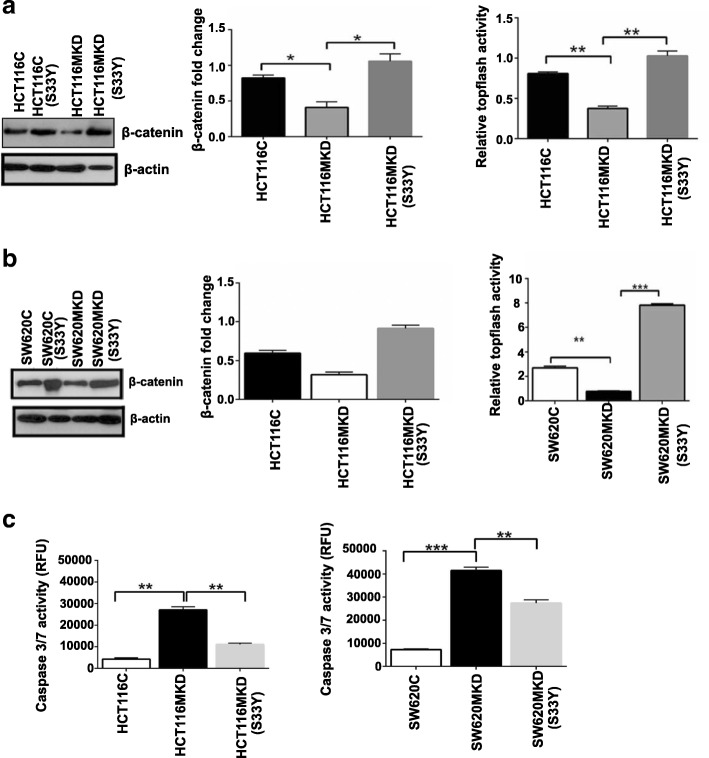
Overexpression of β-catenin-S33Y mutant rescues MASTL knockdown cells from apoptosis. β-catenin-S33Y mutant was transiently overexpressed (48 h) in MASTL knockdown cells and overexpression of activated β-catenin was confirmed by immunoblotting and topflash reporter assay in HCT116 (**a**) and SW620 (**b**) cells. **c** Caspase-3/7 activity as measured by luminescence in HCT116^MKD^ and SW620^MKD^ cells as compared to control cells and modulation due to overexpression of activated form of β-catenin in these cells

Encouraged by these findings, we asked whether effects on apoptosis mediated by MASTL can be ameliorated by simply upregulation of β-catenin expression. We overexpressed a mutant β-catenin (S33Y) construct (which resists proteosomal degradation and thus is highly stable) in both HCT116MKD and SW620MKD cells. Overexpression of β-catenin and its expected effect on promoting Wnt/β-catenin signaling was confirmed by immunoblotting and TOPFlash promoter reporter (Fig. 7a, b). Overexpression of activated β-catenin in MASTL-inhibited cells inhibited apoptosis (40–50%) and levels were similar to controls cells (Fig. 7c) suggesting effects of MASTL on cell viability are mediated by modulating β-catenin expression and activity.

### MASTL expression induces resistance to anti-colon cancer therapy

**Fig. 8:**
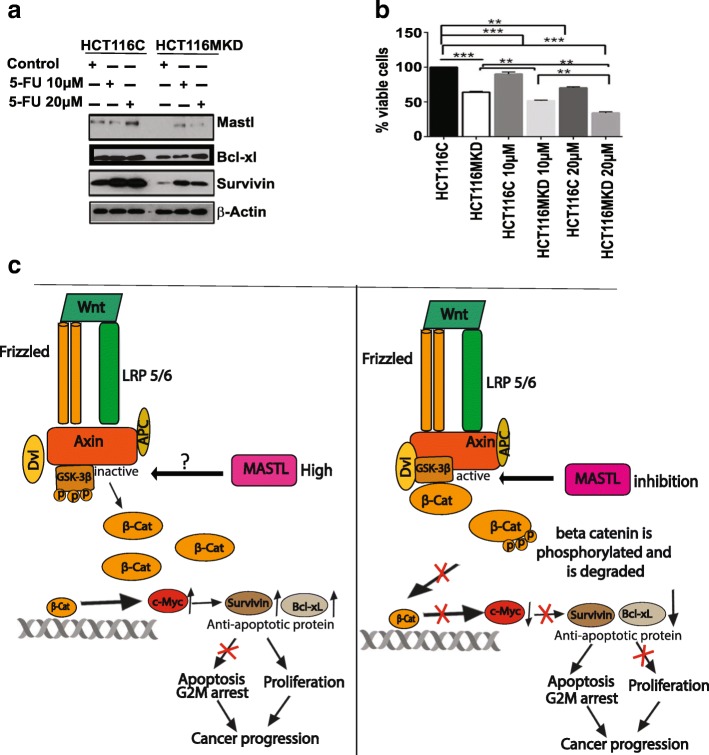
MASTL imparts chemoresistance to 5-FU in colon cancer cell lines. (**a**) HCT116C and HCT116^MKD^ cells were treated with 10 and 20 μM of 5-FU. Western blot analysis demonstrated induction of Survivin and Bcl-xL in control cells. However, inhibition of MASTL inhibited these protein expression even in presence of 5-FU. (**b**) MTT assay in HCT116C and HCT116^MKD^ cells showed significant reduction in viable cells as compared to control treated cells. For graphs, data represent mean ± SD; **, *P* < 0.001; ***, *P* < 0.0001 versus control. (**c**) Model depicting the role of MASTL in regulation of colon cancer progression. When MASTL expression is increased it phosphorylates GSK-3β and inactivates it, thereby β-catenin is not phosphorylated and degraded. The β-catenin then is translocated into nucleus leading to active transcription of its target genes c-Myc, Survivin and Bcl-xL. These cells escape G2M arrest and apoptosis leading to cancer progression. Conversely, in MASTL inhibited cells, GSK-3β is active which then phosphorylates and degrades β-catenin thus preventing its translocation to nucleus and activation of target genes

In head and neck cancer, up-regulation of MASTL expression promotes cancer progression and tumor recurrence after initial cancer therapy [2]. In the light of our data that MASTL expression is directly proportional to the CRC progression, we reasoned that MASTL expression may similarly promote resistance against conventional anti-CRC therapy using 5-FU. To determine the validity of this supposition, control, HCT116^MKD^, and SW620^MKD^ cells were subjected to 5FU-treatment (10 or 20 μM). Immunoblotting using cell lysate prepared from these samples demonstrated significant increases in MASTL expression, along with Survivin and Bcl-xL expressions, suggesting a potential role for these molecules in chemotherapeutic resistance (Fig. 8a). Further analysis showed that the increase in Survivin and Bcl-xL expressions in 5FU-treated control cells was significantly reduced in MASTL-inhibited cells even in the presence of 5FU probably making these cells more sensitive to chemotherapy (Fig. 8a). Similar results were obtained in SW620 cells (Additional file 1: Figure S7).

We then hypothesized that inhibition of MASTL would reduce survival signaling downstream of MASTL and induce chemosensitivity. We again subjected control and MASTL knockdown cells to 5FU-treatment (both cell lines being highly metastatic and chemoresistant). Treatment with 5FU could only induce 25–30% cell death in control HCT116 cells. However, 5- FU treatment was significantly more effective in the same cells in the absence of MASTL expression (HCT116^MKD^), with cell survival significantly reduced (60–70%) (Fig. 8b, *P* < 0.001). Similar results were observed in SW620C and SW620^MKD^ cells. These observations further support a key role for MASTL in resistance to chemotherapeutic agents for colorectal cancer. A postulated model depicting MASTL-dependent regulation of β-catenin to regulate cmyc/Survivin/Bcl-xL expression, associated signaling and cellular functions is presented in Fig. 8c.

## Discussion

The central role of the uncontrolled and/or dysregulated cell division in promoting malignant growth means that targeting the cyclin-dependent kinases (Cdks), key regulators of the cell cycle, is the most desired line of anti-cancer drug development, by university researchers and pharmaceutical companies. Several Cdks, including Polo-like and Aurora kinase, have recently emerged as important regulators of the cell-cycle progression with a causal association to cancer progression [21, 22]. However, attempts to employ these have been hindered primarily by significant side effects associated with killing of the normal cell division that is essential for maintaining function of several organs. In current studies, we identify MASTL as a therapy target in colon tumorigenesis that appears to be highly upregulated in cancer cells, and thus promises minimal toxicity. Our data that this protein not only shows stage-specific increases in CRC patients but negatively associates to patient survival further support its use as a promising anti-cancer therapeutic target. Our additional data that depletion of MASTL expression significantly suppresses chemoresistance in CRC cells against conventional anti-CRC therapy agent 5-FU further highlights its efficacy in effective clinical management of the disease.

Of importance, the MASTL protein has been shown to be critical for mitosis [23]. The MASTL/Greatwall kinase is activated during the G2/M transition due to phosphorylation by cyclin- B-Cdk1, followed by autophosphorylation of the C-terminal activating site. Activation of this kinase, in turn, promotes inhibition of PP2A-B55 through phosphorylation of its substrates, Arpp19 and ENSA [1, 24–26]. This inhibition results in stable phosphorylation of cyclin-B-Cdk1 substrates and mitotic entry. Once mitosis is complete, the cell must exit mitosis, and to do this, the prevailing phosphorylation(s) has to be removed. Removal is suggested to be accomplished by reversing the inhibitory effect of MASTL on phosphatases by PP1 [27]. Our data are well aligned with the understanding of the regulatory role for MASTL in cell cycle regulation in colon cancer cells, given that inhibiting MASTL was sufficient to inhibit cell cycle progression and mitosis. Furthermore, MASTL depleted colon cancer cells demonstrated cell cycle arrest at the G2/M phase and significant increase in apoptosis. The novelty of our studies is in our observation that MASTL regulates Wnt−/β-catenin signaling hyperactivation, critical regulator of colon tumorigenesis, to promote colon carcinogenesis. Mechanistically, based on our data, we postulate that MASTL inhibition leads to activation of Gsk3β, which in turn induces phosphorylation and thus degradation of the oncogenic β-catenin expression. This β-catenin downregulation leads to decrease in cellular content of the c-Myc, Survivin and Bcl-xL, which ultimately leads to apoptotic cell death. Previous studies have shown c-Myc network is required for the majority of Wnt target gene activation following Apc loss within intestinal epithelium [28]. Whether MASTL expression alone is sufficient for this function or its phosphorylation or other activity is also involved remains to be determined.

The role of MASTL in promoting colon cancer is supported by our findings that its expression is markedly increased in colon cancer cells, in transcriptome and protein expression analyses of a large CRC patient cohort, in the cancer genomic atlas (TCGA) database, and in colon tumors that result from mouse model of sporadic or inflammation-induced colon cancer. These findings get strong support from similar upregulation of MASTL through Akt pathway in a recent study using another CRC patient cohort [29]. Moreover, our data from colon cancer cells or xenograft tumor growth assays demonstrate that increased MASTL expression can serve as an independent predictor of poor clinical outcome in colon cancer. Most notably, our studies suggest that normal colonocytes either don’t express MASTL or express it at negligible levels. By contrast, cancer cells demonstrate robust MASTL expression, especially by cells that were highly tumoroigenic and metastatic, including HCT116 and SW620 cells. Thus, inhibition of MASTL expression in these cells negatively affected their ability to grow in soft agar, invade through the matrix, and to induce tumor growth in vivo. Further, inhibition of MASTL expression arrested cell cycle progression in colon cancer cells at the G2/M interphase, and induced apoptosis. Apoptosis, the outcome of a series of regulated cellular events often suppressed in tumors, can induce a variety of genes involved in cell-cycle inhibition by targeting the G2/M checkpoint [30, 31].

Upregulation of β-catenin signaling by its deregulation or mutational activation has been shown to be present in various human cancer types and is associated with cancer progression and metastasis [32–34]. Additionally, it has been observed that levels of β-catenin increases in the S phase, reaching maximum accumulation at late G2/M and further decreases by the next G1 phase [18]. Yet another study demonstrated a plausible mechanism of G2/M cell-cycle arrest and abrogation of the Wnt/β-catenin pathway, using withanolide-D (witha-D), a steroidal lactone in pancreatic adenocarcinoma cells [19]. β-catenin is a critical regulatory molecule of the canonical Wnt-signaling pathway and plays an important role in regulating diverse cellular processes, including cell proliferation, survival, migration, invasion, polarity, differentiation, development, and stem cell self-renewal [35]. c-Myc is a direct target of Wnt/β-catenin-signaling and has been attributed to having roles in chromosomal rearrangement and remodeling through telomeres [36] as well as in G2/M arrest following DNA damage, leading to an inappropriate entry of damaged chromosomes into mitosis [37]. Of interest, c-Myc is aberrantly expressed in 60–80% in CRC and universally implicated in promoting colorectal tumorigenesis [38, 39], including colitis-associated colon adenocarcinomas [40–44] and c-Myc expression confers resistance against 5FU [45–48]. Overexpression of c-Myc is responsible for altering G2/M arrest in aberrant cells, which leads to the entry of damaged chromosomes into mitosis [37], similar to MASTL overexpression. Our findings strongly indicate that MASTL regulate β-catenin expression and cellular localization to modulate its transcription activity and c-Myc expression to regulate colon cancer.

Inactivation of GSK-3β, a primary kinase in the β-catenin multi-protein destruction complex, is frequently found in human cancers. Of note, Akt/GSK-3β phosphorylates β-catenin on conserved serine and threonine residues in its amino terminus to initiate its ubiquitination and subsequent proteasomal degradation [33, 49, 50]. Inactivation of GSK-3β by phosphorylation reduces ubiquitination of β-catenin, resulting in its nuclear accumulation and increased transcriptional activity. In line with this, we detected a decrease in the phosphorylation level of GSK-3β (inactive) that resulted degradation and significant downregulation of total β-catenin protein following inhibition of MASTL expression in HCT116 and SW620 cells. Our results suggest that the MASTL/GSK-3β axis, regulate β-catenin expression. Recent studies using Boolean modeling have also identified Greatwall/MASTL as an important regulator of the Aurora kinase (AURKA) network in neuroblastoma. AURKA overexpression has been shown to mediate pro-tumorigenic functions in addition to mitosis, and drugs aimed at inhibiting its expression to improve anti-cancer therapy are currently under clinical trials [51–53]. Previous studies also demonstrated that AURKA directly binds with GSK-3β, and phosphorylates at Ser9. Whether MASTL associates directly with GSK-3β or indirectly through AURKA, and whether Akt plays a role in this regulation, remains to be determined. GSK-3β has been previously identified as key downstream target of the PI3-kinase/AKT survival signaling pathway [54–56]. It is therefore possible that MASTL regulates GSK-3β phosphorylation through direct interaction, and/or through a MASTL/AKT axis-dependent mechanism.

Another important observation in our studies is that MASTL inhibition renders cells more sensitive to apoptosis that has been induced by 5FU-treatment. Since many anti-cancer drugs result in DNA cross-linking damage, these findings are of high clinical relevance. Our findings suggest that MASTL overexpression can contribute to anti-cancer drug resistance in colon cancer cells by up-regulating Survivin and Bcl-xL expressions. A similar role of MASTL in tumor resistance has been demonstrated in head and neck cancer patients. Of note, MASTL knockdown in recurrent tumor cells re-sensitized their response to cancer therapy in vitro and in vivo*,* and this was similar to our findings in colon cancer cells [2]. MASTL targeting specifically and importantly potentiated non-small cell lung cancer cells to cell death in chemotherapy, while sparing normal cells [1], revealing that MASTL upregulation helps promote cancer progression and tumor recurrence after initial cancer therapy, and strongly supporting MASTL as a promising target of increased therapeutic efficacy of anti-cancer therapies, including anti-CRC therapy.

We show that overexpression of MASTL correlates with colon cancer recurrence and progression. Thus, the inhibition by MASTL of drug-induced cell death may not only account for failure of standard chemotherapy, but may also help explain why MASTL overexpression contributes to the malignant phenotype of colon cancer. The data presented in this study strongly supports a promotive role for MASTL in colon cancer, and the potential association of MASTL with anti-cancer therapy efficacy. Future detailed analyses of a large patient cohort and different publicly available datasets will help confirm the putative role of this protein in prognostic prediction for latent aggressiveness of CRC and resistance to therapy.

## Conclusion

The present study depicts a novel role for MASTL in regulating Wnt/β-catenin signaling to modulate c-Myc and Survivin expression in promoting colon cancer and therapy resistance. Thus understanding the novel functions of MASTL will help in the development of new colon cancer therapeutic approaches.

## Additional file

Additional file 1 **Figure S1.** (**A**) Immunoblotting for normal (IEC-6) and colon cancer cells for MASTL expression. (**B**) Comparison of overall survival in correlation with MASTL expression. Patients were divided into quartiles 1–4 on basis of MASTL expression values. Kaplan-Meier analysis performed, comparing patients in each quartile. Patients with higher MASTL expression have greater overall survival (*P* = 0.09, *n* = 250). **Figure S2.** Inhibition of MASTL expression in SW620 and HCT116 cells. SW620 and HCT116 control and MKD cells were immunostained for MASTL and were co-localized with DAPI. **Figure S3.** Human Oncology array demonstrates downregulation of anti-apoptotic Survivin and Bcl-xL in MASTL-inhibited cells. A-15,16-Bcl-xL, G21,22-Survivin. **Figure S4.** MASTL overexpression induces expression of β-catenin and percentage of viable cells. (**A**) Immunoblot analysis demonstrated induction of β-catenin, Survivin and Bcl-xL in MASTL overexpressing (MOE) SW480 cells. (**B**) Cell viability was also increased in even in presence of 5FU in MASTL overexpressing cells as compared to control cells. **Figure S5.** Correlation between MASTL expression and c-Myc, and BCL2L1. (**A**) MYC expression is significantly upregulated with MASTL expression (*P* < 0.0001, Spearman’s Correlation = 0.4). (**B**) BCL2L1 (Bcl-xL) is significantly upregulated with MASTL expression (*P* = 0.05, Spearman’s correlation = 0.1). **Figure. S6** SW620 control and MASTL knockdown cells treated with 10 and 20 μM of 5-FU. (**A**) Western blot analysis demonstrated induction of β-catenin, Survivin and Bcl-xL in control cells. Inhibition of MASTL inhibited these protein expressions even in presence of 5FU. (**B**) MTT assay and (**C**) caspase activity assay in HCT116 and SW620 control and MASTL knockdown cells showed significant reduction in viable cells as compared to control treated cells. For graphs, data represent mean ± SD; **, *P* < 0.001; ***, *P* < 0.0001 versus control. (PDF 767 kb)

## Abbreviations

APC: adenomatous polyposis coli *(APC)*
CAC: Colitis associated cancer
CDK: cyclin dependent kinase
CRC: Colorectal Cancer
MASTL: Microtubule associated serine threonine like kinase
