# Supplementary material for: MASTL induces Colon Cancer progression and Chemoresistance by promoting Wnt/β-catenin signaling
Source: Mol Cancer. 2018 Aug 1;17:111. doi: 10.1186/s12943-018-0848-3 (PMC6090950; doi:10.1186/s12943-018-0848-3)
Supplement: Supplementary file 1 — Figure S1. (A) Immunoblotting for normal (IEC-6) and colon cancer cells for MASTL expression. (B) Comparison of overall survival in correlation with MASTL expression. Patients were divided into quartiles 1–4 on basis of MASTL expression values. Kaplan-Meier analysis performed, comparing patients in each quartile. Patients with higher MASTL expression have greater overall survival (P = 0.09, n = 250). Figure S2. Inhibition of MASTL expression in SW620 and HCT116 cells. SW620 and HCT116 control and MKD cells were immunostained for MASTL and were co-localized with DAPI. Figure S3. Human Oncology array demonstrates downregulation of anti-apoptotic Survivin and Bcl-xL in MASTL-inhibited cells. A-15,16-Bcl-xL, G21,22-Survivin. Figure S4. MASTL overexpression induces expression of β-catenin and percentage of viable cells. (A) Immunoblot analysis demonstrated induction of β-catenin, Survivin and Bcl-xL in MASTL overexpressing (MOE) SW480 cells. (B) Cell viability was also increased in even in presence of 5FU in MASTL overexpressing cells as compared to control cells. Figure S5. Correlation between MASTL expression and c-Myc, and BCL2L1. (A) MYC expression is significantly upregulated with MASTL expression (P < 0.0001, Spearman’s Correlation = 0.4). (B) BCL2L1 (Bcl-xL) is significantly upregulated with MASTL expression (P = 0.05, Spearman’s correlation = 0.1). Figure. S6 SW620 control and MASTL knockdown cells treated with 10 and 20 μM of 5-FU. (A) Western blot analysis demonstrated induction of β-catenin, Survivin and Bcl-xL in control cells. Inhibition of MASTL inhibited these protein expressions even in presence of 5FU. (B) MTT assay and (C) caspase activity assay in HCT116 and SW620 control and MASTL knockdown cells showed significant reduction in viable cells as compared to control treated cells. For graphs, data represent mean ± SD; **, P < 0.001; ***, P < 0.0001 versus control. (PDF 767 kb) [file 12943_2018_848_MOESM1_ESM.pdf]

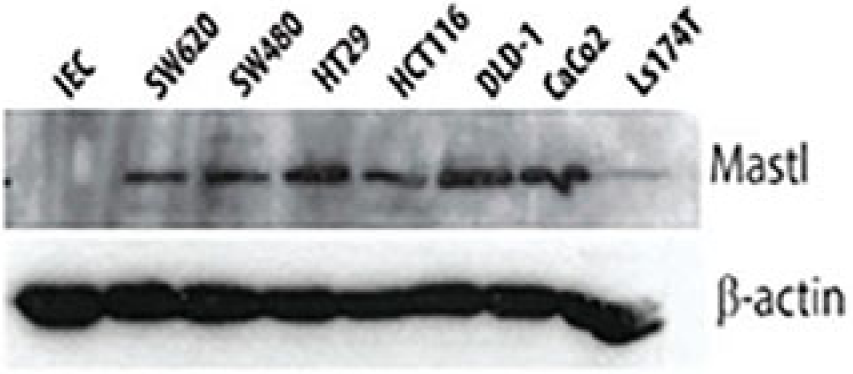
IEC
SW620
SW480
HT29
HCT116
DLD-1
CaCo2
Ls174T
Mastl
β-actin

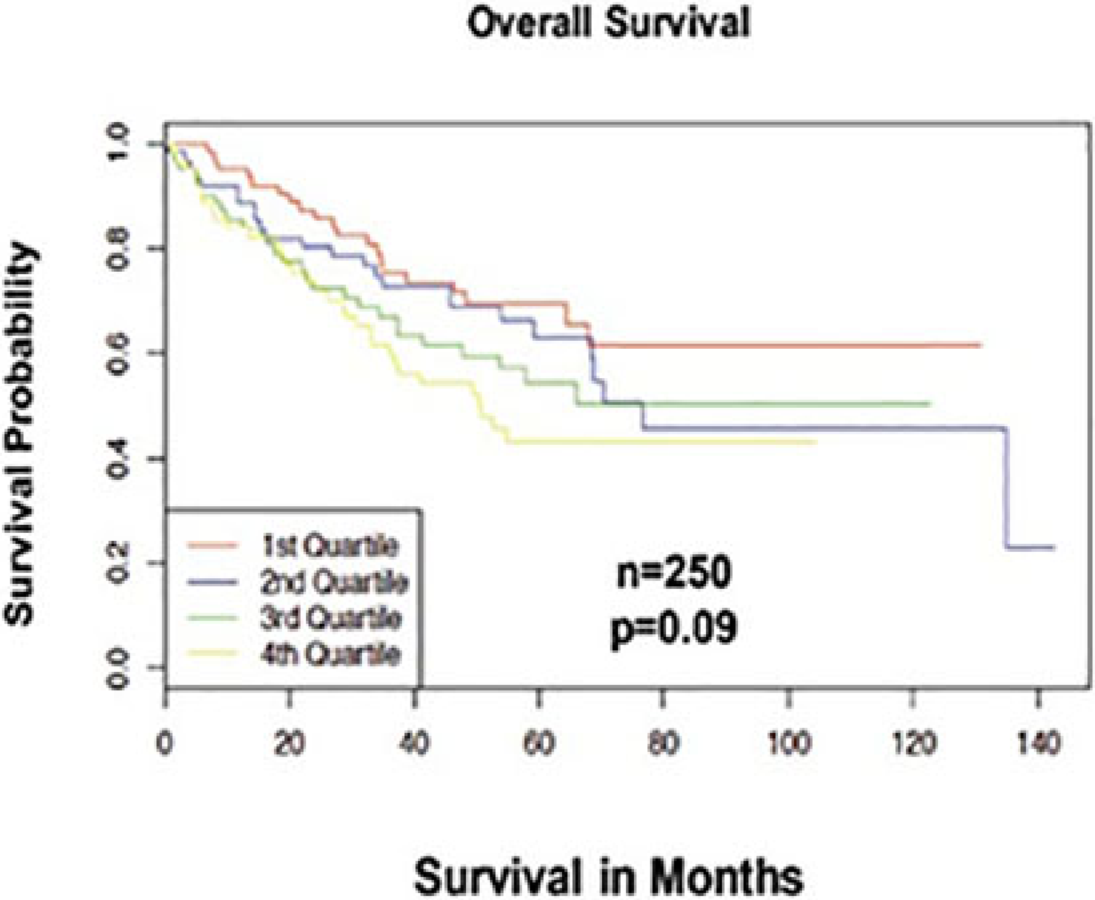
Overall Survival
Survival Probability
1.0
0.8
0.6
0.4
0.2
0.0
1st Quartile
2nd Quartile
3rd Quartile
4th Quartile
n=250
p=0.09
0
20
40
60
80
100
120
140
Survival in Months

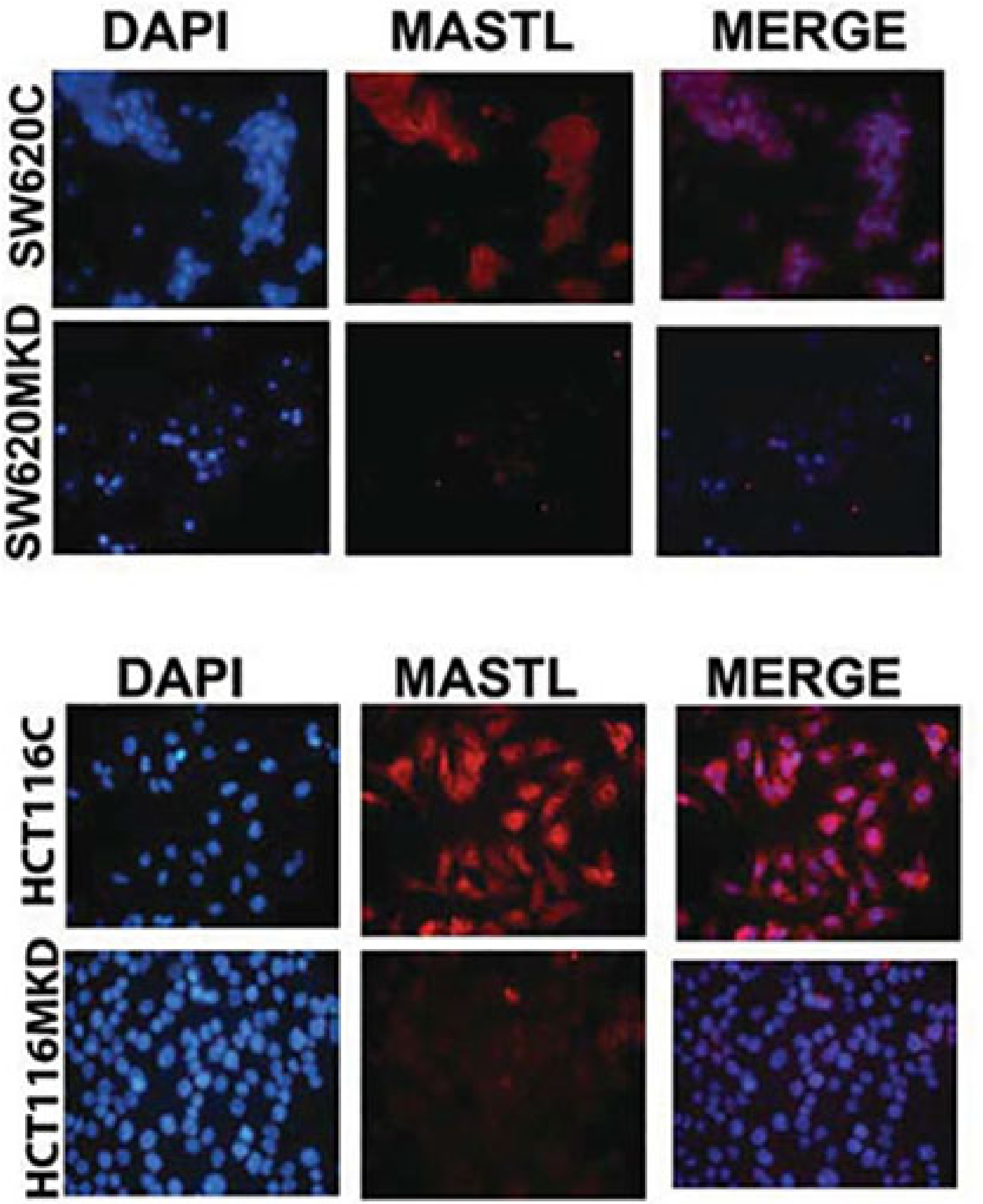
DAPI
MASTL
MERGE
SW620C
SW620MKD
DAPI
MASTL
MERGE
HCT116C
HCT116MKD

A

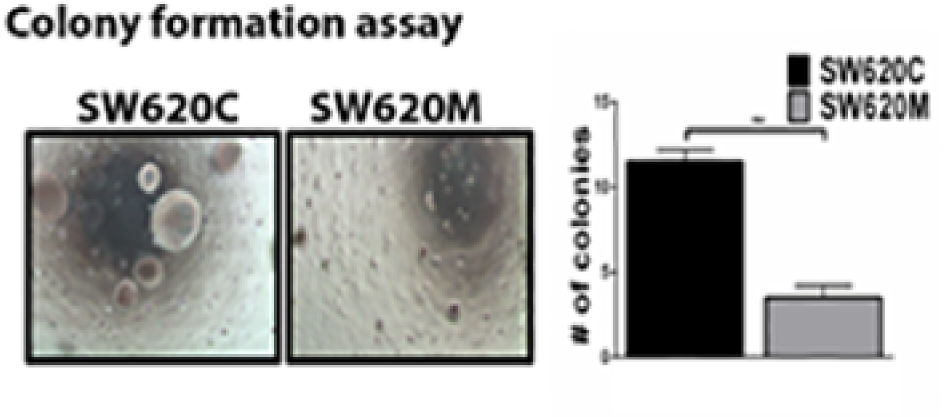
Colony formation assay
SW620C
SW620M
SW620C
SW620M
# of colonies
15
10
5
0

B

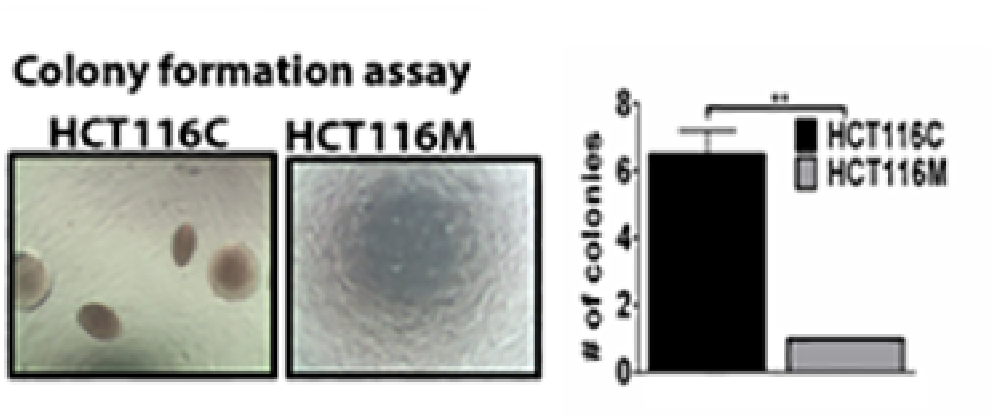
Colony formation assay
HCT116C
HCT116M
HCT116C
HCT116M
# of colonies
8
6
4
2
0
**

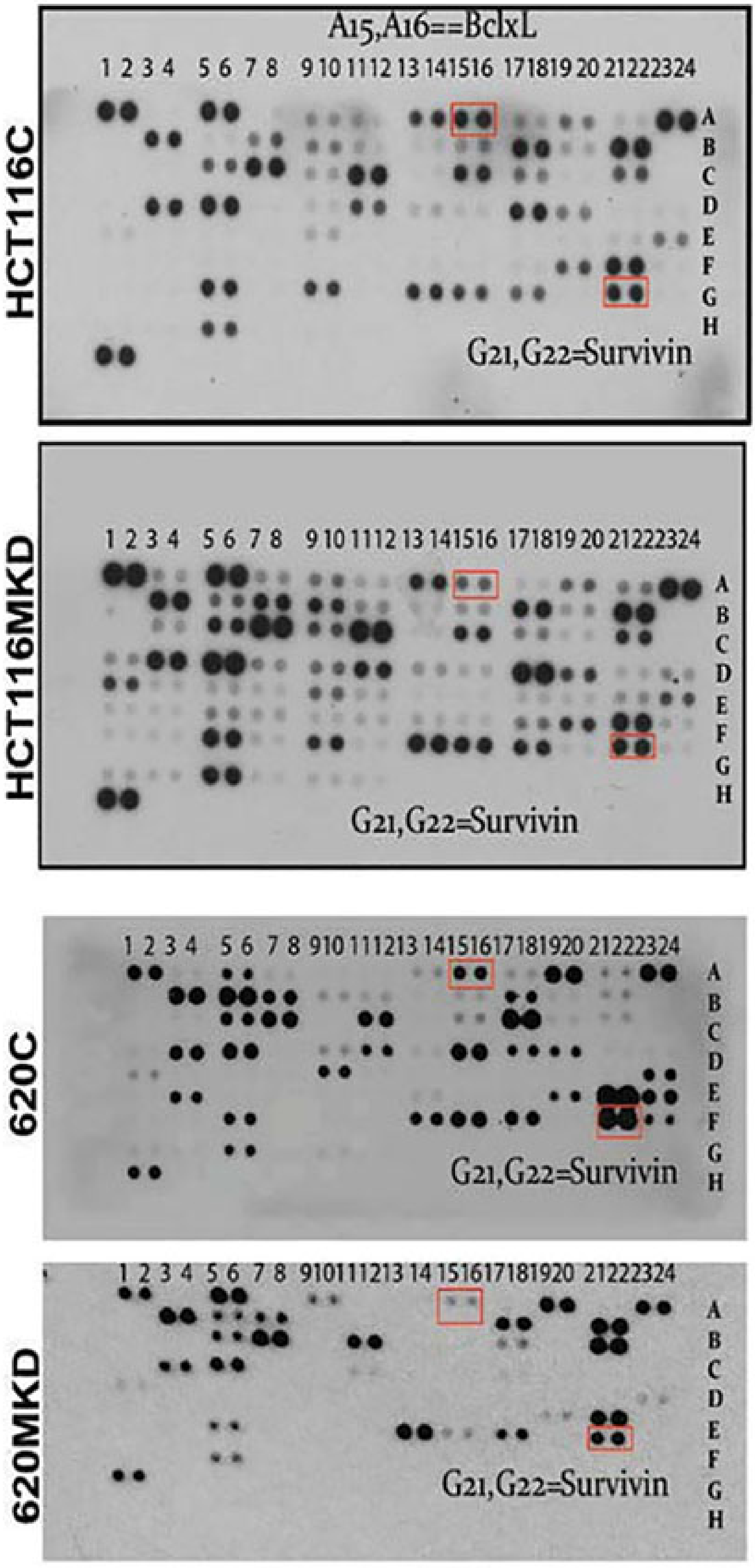
HCT116C
A15,A16==BclxL
1 2 3 4 5 6 7 8 9 10 11 12 13 14 15 16 17 18 19 20 21 22 23 24
A B C D E F G H
G21,G22=Survivin
HCT116MKD
1 2 3 4 5 6 7 8 9 10 11 12 13 14 15 16 17 18 19 20 21 22 23 24
A B C D E F G H
G21,G22=Survivin
620C
1 2 3 4 5 6 7 8 9 10 11 12 13 14 15 16 17 18 19 20 21 22 23 24
A B C D E F G H
G21,G22=Survivin
620MKD
1 2 3 4 5 6 7 8 9 10 11 12 13 14 15 16 17 18 19 20 21 22 23 24
A B C D E F G H
G21,G22=Survivin

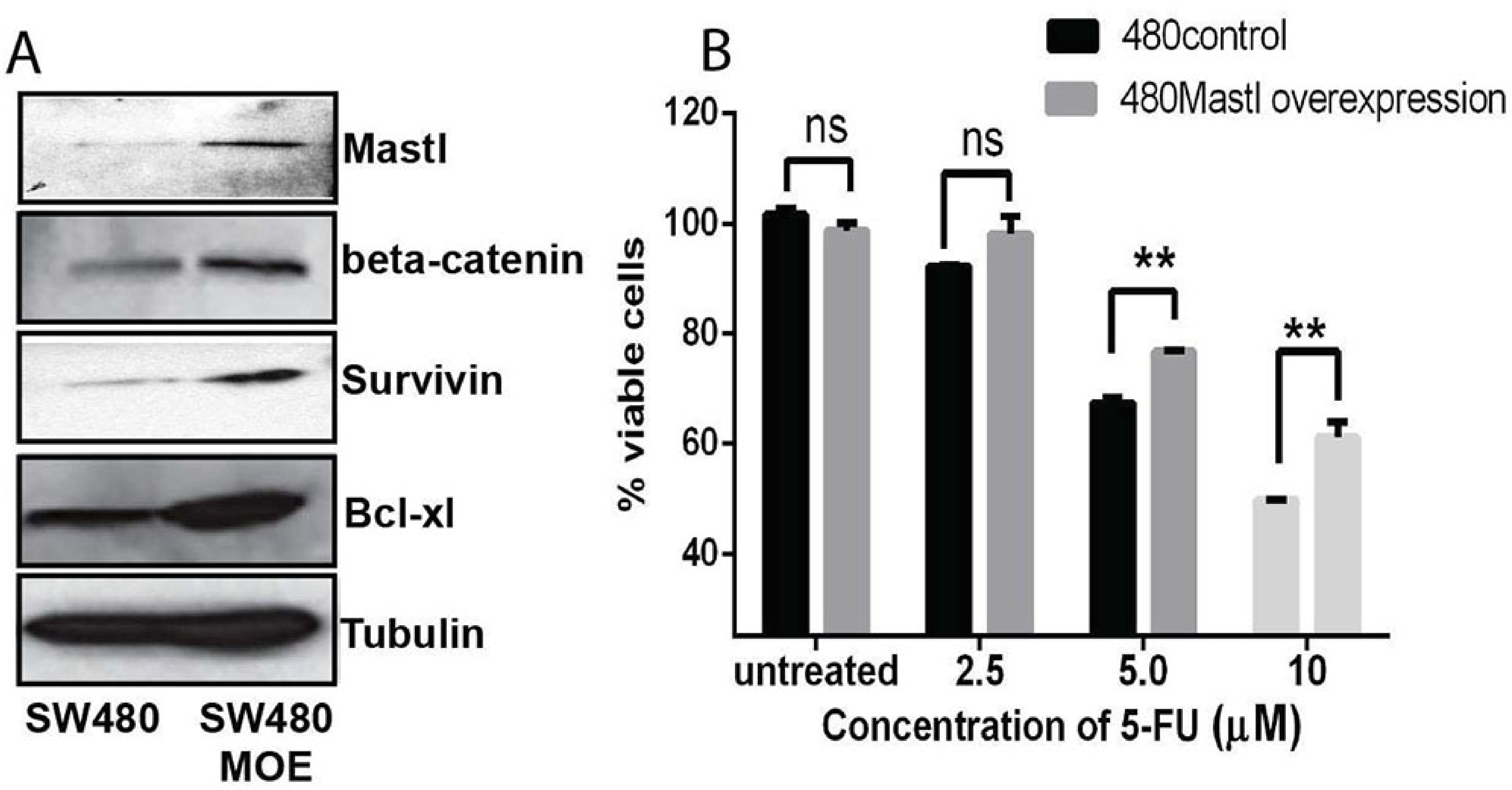
A
Mastl
beta-catenin
Survivin
Bcl-xl
Tubulin
SW480
SW480
MOE
B
480control
480Mastl overexpression
% viable cells
120
100
80
60
40
ns
ns
**
**
untreated
2.5
5.0
10
Concentration of 5-FU (μM)

MYC

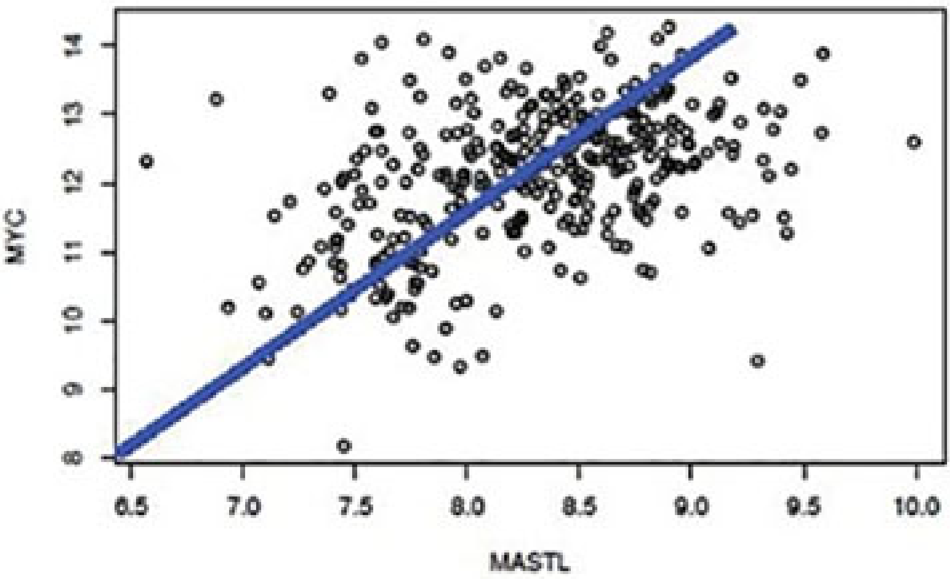

p<0.0001
Spearman's correlation: 0.4

BCL2L1

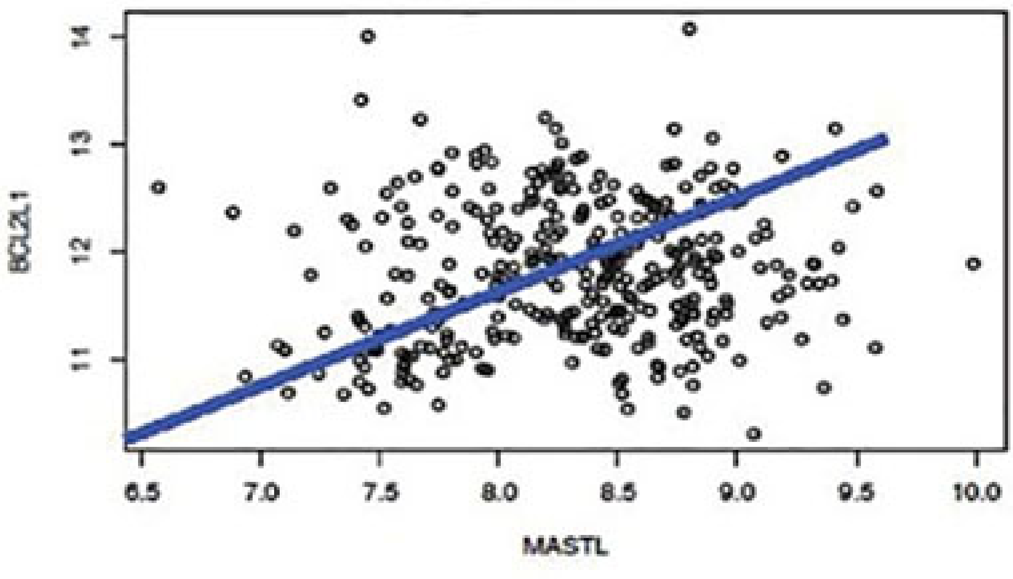

p=0.05
Spearman's correlation: 0.1

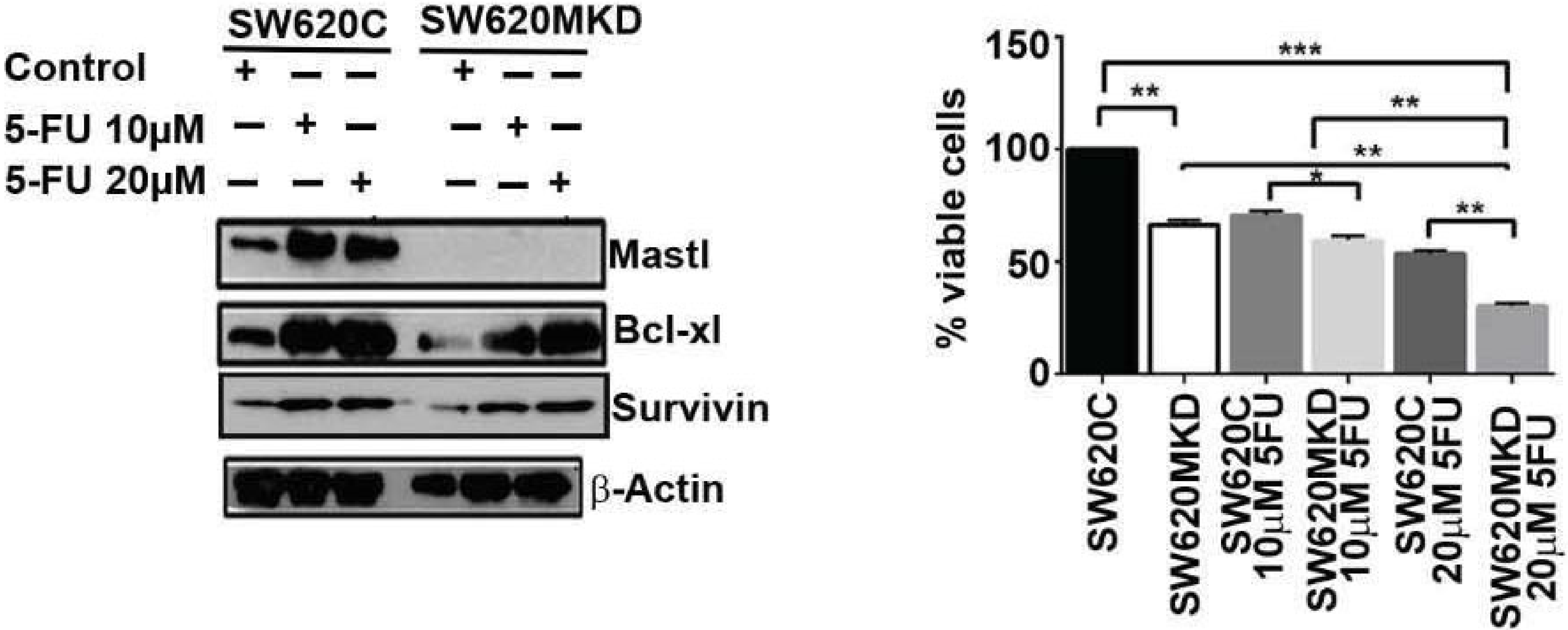

SW620C
SW620MKD
Control
5-FU 10μM
5-FU 20μM
Mastl
Bcl-xl
Survivin
β-Actin
% viable cells
150
100
50
0
***
**
**
**
*
**
SW620C
SW620MKD
SW620C 10μM 5FU
SW620MKD 10μM 5FU
SW620C 20μM 5FU
SW620MKD 20μM 5FU
